# A Novel Detection Scheme for High-Resolution Two-Dimensional Spin-Echo Correlated Spectra in Inhomogeneous Fields

**DOI:** 10.1371/journal.pone.0084032

**Published:** 2014-01-02

**Authors:** Yuqing Huang, Zhiyong Zhang, Shuhui Cai, Zhong Chen

**Affiliations:** Department of Electronic Science, Fujian Provincial Key Laboratory of Plasma and Magnetic Resonance, State Key Laboratory of Physical Chemistry of Solid Surfaces, Xiamen University, Xiamen, China; Wake Forest University, United States of America

## Abstract

**Background:**

Two-dimensional (2D) nuclear magnetic resonance (NMR) spectroscopy is a powerful and non-invasive tool for the analysis of molecular structures, conformations, and dynamics. However, the inhomogeneity of magnetic fields experienced by samples will destroy spectral information and hinder spectral analysis. In this study, a new pulse sequence is proposed based on the modulation of distant dipolar field to recover high-resolution 2D spin-echo correlated spectroscopy (SECSY) from inhomogeneous fields.

**Method and Material:**

By using the new sequence, the correlation information between coupled spins and the *J* coupled information with straightforward multiplet patterns can be obtained free from inhomogeneous line broadening. In addition, the new sequence is also suitable for non-*J* coupled spin systems. Although three-dimensional acquisition is needed, the evolution of indirect detection dimensions is carefully designed and the ultrafast acquisition scheme is utilized to improve the acquisition efficiency. A chemical solution of butyl methacrylate (C_8_H_14_O_2_) in DMSO (C_2_H_6_SO) in a deshimmed magnetic field was tested to demonstrate the implementation details of the new sequence. The performance of the new sequence relative to the conventional SECSY sequence was shown by using an aqueous solution of main brain metabolites in a deshimmed magnetic field.

**Conclusion:**

The results reveal that the new sequence provides an attractive way to eliminate the inhomogeneous spectral line broadening for the spin-echo correlated spectrum and is a promising tool for the study of metabolites in metabonomics, even for the applications on *in vivo* and *in situ* high-resolution 2D NMR spectroscopy.

## Introduction

Two-dimensional (2D) nuclear magnetic resonance (NMR) spectroscopy is a powerful and non-invasive tool for the analysis of molecular structures, conformations, and dynamics [1]–[3]. Spectral overlap in one-dimensional (1D) NMR spectra of complex samples can be alleviated by using 2D techniques. Correlation spectroscopy (COSY) [4] and *J*-resolved spectroscopy (*J*RES) [5] are the pioneers and significant 2D NMR techniques. Combining the merits of these two techniques, spin-echo correlated spectroscopy (SECSY) [6] was proposed. SECSY can be intuitively considered as a modified version of the *J*RES, where the central 180° pulse is replaced by a 90° pulse for the coherence transfers. Besides the insensitivity to the field inhomogeneity along the indirect dimension as found in *J*RES, a SECSY spectrum holds the same information as a COSY spectrum. For a SECSY spectrum, the diagonal peaks, representing the inequivalent nuclei, lie at the F1 = 0 line because the chemical shift evolution is refocused in the F1 dimension, and the cross peaks, indicating scalar coupling between coupled resonances, occur in pairs along a line which forms an angle of 135° with the F2 axis. Spin connectivity between coupled resonances can be obtained by matching the parallelograms with their corresponding diagonals lying at the F1 = 0 line. SECSY allows a smaller data matrix than COSY by substituting the original “whole chemical shifts” with “chemical shift differences” of coupled spins in the F1 dimension, which yields a special spectral presentation. SECSY has been widely used in NMR fields. For example, it has been used in the detection of carbohydrate peptide linkage [7]. The SECSY experiment has been used for the interpretation of magic angle spinning NMR data of on-resin products [8]. In addition, the 2D localized SECSY technique was performed on a whole body MRI/MRS scanner to investigate glutamate/glutamine, NAA, and other *J*-coupled metabolites in human brain [9].

However, the SECSY spectral quality is generally demolished by inhomogeneous line broadening caused by variations of external static magnetic field or magnetic susceptibilities in the sample. The spin-echo scheme in SECSY can remove the inhomogeneous line broadening in the F1 dimension, but the influence of field inhomogeneity remains in the F2 dimension and results in the loss of exact chemical shift information and the peak overlap along F1 = 0. Due to the half-size of *J* coupling splitting along the F1 dimension and the complicated multiplet patterns of peaks in the SECSY spectrum, it is ambiguous for the recognition of exact *J* coupling information, especially in inhomogeneous fields. Therefore it will be useful to design a new SECSY version with straightforward multiplet patterns in inhomogeneous fields.

The distant dipolar field (DDF) effect has long been recognized and applied in many NMR fields, ranging from magnetic resonance imaging (MRI) [10]–[13] to NMR spectroscopy [14]–[19]. Generally, a concentrated proton component should be contained in the testing sample for the generation of DDF. This is easy to satisfy since chemical or biological samples usually contain a concentrated solvent, such as water. The modulation of DDF results in intermolecular multiple-quantum coherence (iMQC) signals. The iMQC signals generally come from solvent-solute spin pairs which are physically close (typical distance 10∼100 µm) and dipolar coupled to each other [20]. The field inhomogeneity beyond the distant dipolar correlation distance will not affect the signals. Therefore, it is attractive to apply the DDF effect for high-resolution spectra in inhomogeneous fields.

In this study, a new pulse sequence named DDF-SECSY is designed for high-resolution 2D SECSY in inhomogeneous fields. Similar to the iMQC 1D high-resolution approaches *via* 2D acquisition [14], three-dimensional (3D) acquisition is required for the DDF-SECSY sequence and high-resolution 2D SECSY information can be recovered after discarding one dimension while preserving the other two dimensions free from field inhomogeneous effects. The manipulation of indirect evolution periods with corresponding 3D data shearing process is applied to effectively shorten 3D acquisition time and achieve straightforward multiplet patterns of *J* couplings. To further shorten acquisition time, an alternative acquisition scheme, i.e. DDF-SECSY in combination with ultrafast acquisition technique [21], is supplied by substituting indirect-dimension *t*_2_ increments with a single scan in acquisition dimension.

## Methods

**Figure 1:**
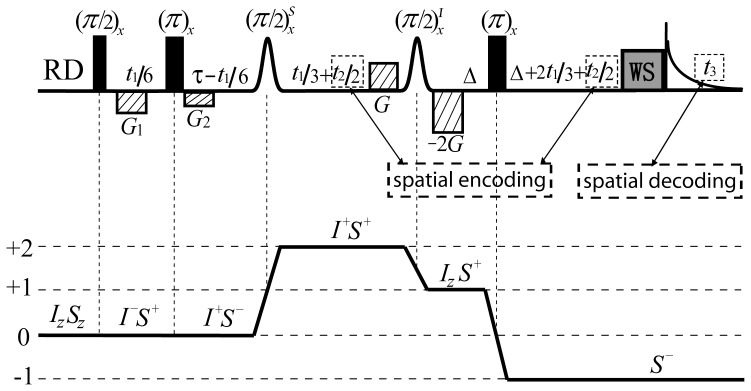
The DDF-SECSY sequence for high-resolution 2D SECSY spectra in inhomogeneous fields *via* 3D acquisition. Full vertical bar stands for non-selective RF pulses, Gauss-shaped pulses are selective RF pulses, dash rectangles represent coherence selection gradients, “WS” is the spare module for solvent suppression. An optional ultrafast acquisition scheme is supplied by substituting the indirect detection period *t*_2_ and direct detection period *t*_3_ with spatially encoded and spatially decoded schemes, respectively, shown as the dotted-box modules. The coherence transfer pathway is given and the raising and lowering operators are applied to show the coherence states of *I* and *S* spins.

The DDF-SECSY pulse sequence is shown in Fig. 1. Four linear coherence selection gradients (CSGs) are applied along the *z* direction to select the coherence transfer pathway 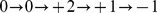, in which the first two CSGs, *G*_1_ and *G*_2_, are used to dephase all the coherence orders except for zero-quantum coherence, and the third and fourth CSGs with an area ratio of 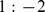 form a double-quantum filter to eliminate the residual conventional single-quantum coherence (SQC) signals. Two indirect evolution periods *t*_1_ and *t*_2_ are set for the desired 3D data in such coherence transfer pathway. The *t*_1_ evolution period is composed of four parts: *t*_1_/6, 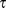-*t*_1_/6, *t*_1_/3, 2*t*_1_/3. The first two parts are zero-quantum coherence evolutions and they form a constant-time (CT) scheme in which the minimum value for 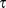 is 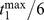, while the last two parts form an echo scheme for the double-quantum coherence evolution. For this manipulation of *t*_1_ evolution period, field inhomogeneous effects can be eliminated along the F1 dimension and the multiplet patterns of *J* coupling are simplified. The *t*_2_ evolution period is equally divided into two parts to form a delayed acquisition scheme with direct detection period *t*_3_, so only half of the range of field inhomogeneity occupies the F2 dimension. This provides a substantial decrease of the F2 spectral width needed to be sampled and greatly shortens the experimental time. To further improve acquisition efficiency, an ultrafast acquisition option was designed by substituting the indirect detection period *t*_2_ and direct detection period *t*_3_ by a constant-time spatially encoded scheme and echo planar imaging (EPI) spatially decoded scheme [22] respectively, shown by the dotted-box modules in Fig. 1. A water suppression (WS) module [23] was appended at the end of the sequence to further suppress the residual solvent signal.

In present work, the DDF treatments together with the product operator formalism [24] are employed to deduce the analytical expression of signals from the DDF-SECSY sequence. Without loss of generality, a homogeneous liquid mixture consisting of *S* and *I* components is taken as an example. *S* is an AX spin-1/2 system (including 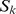 and 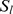 spins with a scalar coupling constant 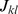) and *I* is a single spin-1/2 system. It is assumed that *I* (corresponding to solvent) is abundant and *S* (corresponding to solute) is either abundant or dilute. Let 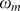 be the frequency offset of spin 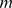 (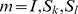) in the rotating frame in the absence of field inhomogeneity. The magnetic field is assumed to be only inhomogeneous along the *z* axis, and 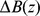 is the field inhomogeneity at position *z*. For simplification, the effects of radiation damping, diffusion, relaxation, and intermolecular NOE are ignored. The module of water suppression right before acquisition only acts to suppress the residual solvent signal and does not influence the desired solute signals, therefore it was ignored in the following derivation. If the magnetization is fully modulated and varies only in one direction, such as the *z*-direction, the DDF is localized, and an exact theoretical expression for the DDF-SECSY signals can be obtained [20]. As we are interested in the evolution of magnetization, we only consider the reduced density operator in the following derivation. For the *I*+*S* spin system discussed herein, the reduced density operator at the thermal equilibrium state with the high-temperature approximation can be given by
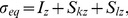
(1)where the Boltzmann factor has been omitted for clarity; 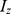, 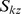, and 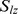 represent the longitudinal components of *I*, *S_k_*, and *S_l_* spins, respectively. Since the analytical expression of the signal from the *S_k_* spin are similar to that of the signal from the *S_l_* spin in an AX spin-1/2 system, only the signal from the *S_k_* spin is considered in the following deduction. When the DDF-SECSY sequence is applied, the *I* spin magnetization contains the spatially modulated longitudinal component, 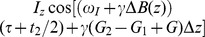, which is generated after the solvent-selective 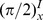 RF pulse. According to the DDF treatment, the effective DDF results from the spatially modulated longitudinal *I* spin magnetization. The effective DDFs 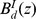 and 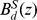 experienced by the *I* and *S* spins can be written as
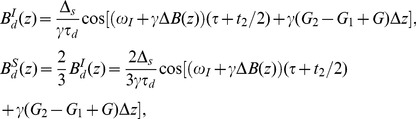
(2)where 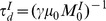 is the dipolar demagnetizing time of *I* spin, in which 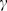 is the gyromagnetic ratio, 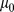 is the vacuum magnetic permeability, and 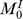 is the equilibrium magnetization per unit volume of *I* spin; 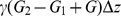 is the dephasing angle at position *z* due to the CSGs, in which *G*_1_, *G*_2_, *G*, and *δ* are strength and duration of the CSGs, respectively; 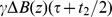 is the dephasing angle of spins at position *z* due to field inhomogeneity; 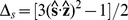, in which 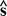 is the unit vector along the CSG direction, and 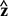 is the unit vector along the direction of static magnetic field. Since the pulse field gradients are oriented along the *z* direction, i.e. 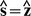, we have 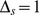. Under the modulation of DDFs, the observable transverse spin density during the acquisition period is
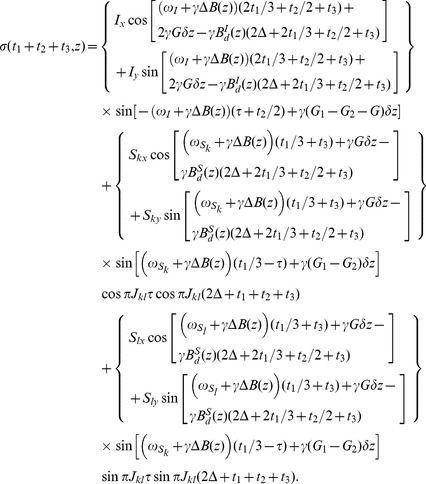
(3)


When the quadrature detection is applied, the total complex transverse magnetization at position *z* can be obtained as
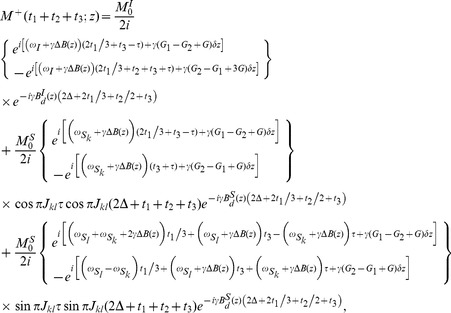
(4)where 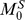 is the equilibrium magnetization per unit volume of *S* spin. Using the Bessel function expansion 
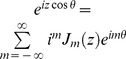
, in which 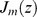 is the Bessel function of the order *m* and *z* is the argument for the Bessel function [25], the terms involving DDF effects in Eq. (4), 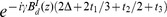 and 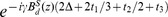, can be expanded and rearranged to yield
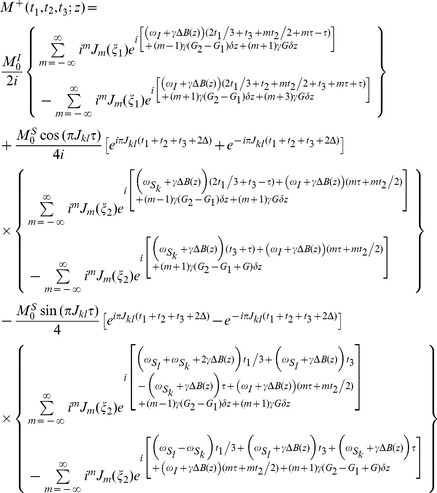
(5)where the order of Bessel function *m* should be integer for all the terms; the arguments of the Bessel functions 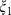 and 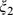 are given by 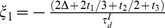 and 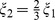. To evaluate the detectable signals from the whole sample, an average of the complex magnetization over all *z* positions should be taken. The dipolar correlation distance for our sequence is 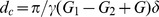. If this distance is much smaller than the sample size, the spatial averaging across the sample causes the signals to vanish unless *m*−1 = 0 and *m*+1 = 0 synchronously for the first term, *m*+1 = 0 and *m*+3 = 0 synchronously for the second term, *m*−1 = 0 and *m*+1 = 0 synchronously for the third term, *m*+1 = 0 for the fourth term, *m*−1 = 0 and *m*+1 = 0 synchronously for the fifth term, and *m*+1 = 0 for the sixth term in Eq. (5), which are independent of the absolute position in the sample [26]. Since no integer satisfies the requirements for *m* in the first and second terms in Eq. (5), they disappear after the spatial averaging, which shows that the solvent signals are in principle eliminated by the designed coherence selection. Similarly, no integer satisfies the requirements for *m* in the third and fifth terms in Eq. (5), therefore these contributions are also removed. When 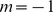 for the fourth and sixth terms, the observable signals are given by:
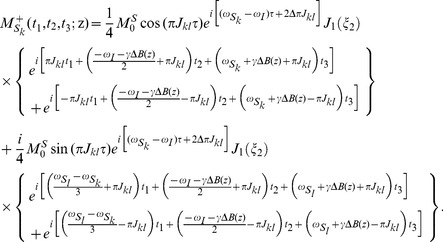
(6)


Equation (6) provides a quantitative expression of the 3D signals for an *S_k_* spin obtained from the DDF-SECSY. The signals split into four terms due to the *J* coupling and coherence transfers, the first two terms represent the diagonal peaks for *S_k_* spin, located at 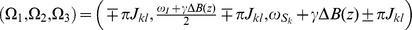, while the last two terms represent the cross peaks between the coupled *S_k_* and *S_l_* spins, located at 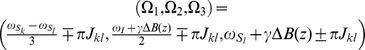. When the spectrometer reference frequency is set to the resonant frequency of *I* spin, i.e. 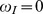, only half of inhomogeneous broadened line-width plus maximal *J* coupling constant is needed to be covered along the F2 dimension. The acquisition time for a 3D DDF-SECSY spectrum relies on the value of 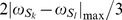 along the F1 dimension and the range of 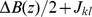 along the F2 dimension. This time is significantly shorter than the time required by conventional 3D acquisition. Note that the inhomogeneous line broadening effect has been removed in the F1 dimension. However, the F2 and F3 dimensions are still subjected to the influence of field inhomogeneity, and a high-resolution 2D spectrum cannot be extracted from this original 3D data. Fortunately, a shearing process of the F2–F3 planes along the F3 axis can be performed to eliminate the inhomogeneous broadening along the F3 dimension, i.e. 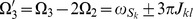. The location of cross peak in the sheared 3D spectrum becomes 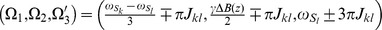. A high-resolution 2D SECSY can be obtained by projecting the sheared 3D spectrum onto the F1–F3 plane. The analytical expression for the resulting 2D projection spectrum is given by:
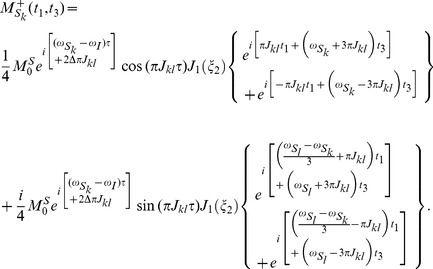
(7)


**Figure 2:**
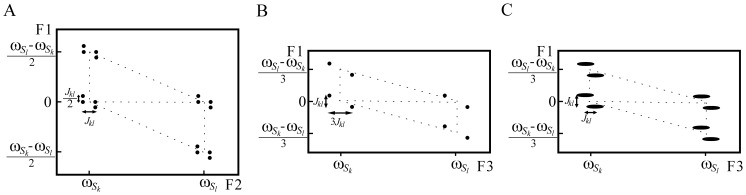
Schematic SECSY spectra for an AX spin system: (A) conventional SECSY in homogeneous fields; (B) and (C) DDF-SECSY and DDF-SECSY with the ultrafast acquisition scheme recovered from inhomogeneous fields.

Equation (7) provides a quantitative expression of the diagonal and cross peaks in the high-resolution 2D DDF-SECSY projection spectrum from the 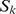 spin. The diagonal peak appears at 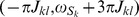 and 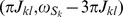, which indicates that the diagonal peak is centered at 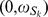 and split into two peaks apart with 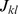 along the F1 axis and 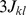 along the F3 axis (Fig. 2B). This multiplet pattern is simpler than that in conventional SECSY spectrum in which four peaks apart with 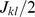 along the F1 axis and 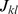 along the F2 axis exist (Fig. 2A). For complex spin systems, there are often several couplings acting on a given proton, which makes the *J*-splitting pattern complicated and difficult to assign in conventional SECSY and even in COSY spectrum. The DDF-SECSY provides a unique way for the interpretation of diagonal and cross peaks via simplified multiplet patterns. The *J* coupling constant is magnified by a scale factor of 3 in the F3 dimension, therefore the DDF-SECSY sequence is more suitable for the measurement of spin systems with small *J* coupling constants. The cross peak of 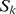 spin in the 2D DDF-SECSY projection spectrum is centered at 
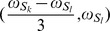
 and holds the same multiplet patterns as the diagonal peak. Compared to the chemical shift difference 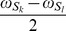 obtained from a conventional SECSY experiment, 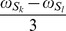 is obtained in the 2D DDF-SECSY spectrum, which requires smaller spectral width. In addition, it can be noticed from Eq. (7) that the intensity of diagonal peak is related to 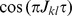 while that of cross peak is related to 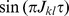, hence the extreme intensity difference between diagonal and cross peaks in conventional SECSY can be avoided by a reasonable setting of 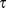, which is more comprehensive for the SECSY information.

When the optional spatially encoded and decoded modules are applied in the F2 and F3 dimensions of the DDF-SECSY sequence, the acquisition time for a 3D DDF-SECSY spectrum only relies on the value of 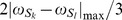 along the F1 dimension. Since the spatially encoded period in *t*_2_ is constant, there is no *J* coupling information along the F2 dimension. After the same shearing process on the original acquisition data and 2D projection of the reconstructed 3D data, the diagonal and cross peaks in the resulting projection spectrum will locate at 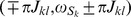 and 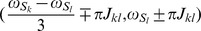, respectively, and the original *J* coupling information is preserved in both the F1 and F3 dimensions (Fig. 2C). However, due to the intrinsic resolution defect of the ultrafast acquisition scheme, the spectral resolution along the ultrafast dimension (F3) is generally lower than that along the F1 dimension in the resulting spectrum [27].

The above theoretical conclusions are derived from an AX spin system, it should also hold for other complicated *J* coupled and non-*J* coupled spin systems since the DDF originated from the solvent spin acts on all the solute spins evenly and is independent of the *J* coupling evolutions and coherence transfer. Experiments were carried out to verify the theoretical analysis.

## Experiments

### Experiments on a chemical solution

All experiments were performed at 298 K using a Varian NMR System 500 MHz spectrometer, equipped with a 5 mm ^1^H {^15^N-^31^P} XYZ indirect detection probe with 3D gradient coils. A solution of butyl methacrylate (C_8_H_14_O_2_) in DMSO (C_2_H_6_SO) with a molar ratio of 1∶8 was used to demonstrate the implementation details of the proposed sequence shown in Fig. 1. The magnetic field was intentionally deshimmed to produce broad peaks with 150 Hz line-width. In this inhomogeneous field, the 2D SECSY spectra were acquired using the new sequence and conventional sequence. A conventional 2D SECSY experiment in a well-shimmed field (specify linewidth) was obtained for comparison. The conventional 2D SECSY experiments were performed with 1.5 s repetition time and two transients, 2048×4512 points were acquired with spectral widths of 2300 Hz×3800 Hz (F1×F2) in 1.7 h. For the DDF-SECSY sequence in inhomogeneous field, the selective pulse for solute spins was constitutive of a 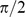 hard RF pulse and a solvent selective 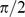 Gaussian pulse with an opposite phase. The width of the 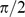 hard RF pulses was set to 10 µs and the width of the selective 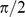 Gaussian pulse for solvent spins was 5.5 ms. The parameters of the CSGs were *G*_1_ = 0.09 T/m, *G*_2_ = 0.04 T/m, *G* = 0.14 T/m, and 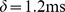. The parameters of the gradient pulses in the WS module were *G*_3_ = 0.07 T/m, *G*_4_ = 0.18 T/m, and 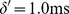. No phase cycling was applied in the DDF-SECSY experiments. The pulse repetition time was 1.0 s, the constant-time 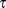 was 120 ms and the echo time (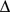) was 50 ms. 800×30×928 points were acquired with spectral widths of 1450 Hz×120 Hz×3800 Hz (F1×F2×F3) in 6.66 h. For the DDF-SECSY with ultrafast acquisition scheme, a pair of *π* chirped pulses with encoding gradient forms the spatial encoding module, in which the spatially encoded gradient 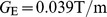 and its duration is 10 ms. During the detection period, the signals were decoded by an EPI acquisition module with the amplitude of spatially decoded gradient 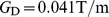 and its duration 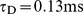. Both spatial encoding and decoding gradients were applied along *z* direction. Except for the ultrafast acquisition parameters, all the experimental parameters were kept the same as those for DDF-SECSY. The ultrafast dimensions (F2×F3) with spectral widths of 200 Hz×3800 Hz were acquired. The F1 dimension with a spectral width of 1450 Hz was acquired with 800 *t*_1_ increments and two transients. The total acquisition time was about 26.7 min. All the DDF-SECSY 3D data were processed using our custom-written program with Matlab 7.8 (The Math Works Inc.).

### Experiments on a brain phantom

To test the ability of the proposed sequence, an aqueous solution of brain metabolites containing 50 mM N-acetyl-DL-aspartic acid (NAA), 45 mM creatine hydrate (Cr), 40 mM choline chloride (Cho), 40 mM L-glutamic acid (Glu), 35 mM GABA, 45 mM myo-inositol (m-Ins), 20 mM taurine (Tau), 40 mM DL-lactic acid (Lac), and 45 mM alanine (Ala) was studied in an inhomogeneous field with 200 Hz line-width. The conventional SECSY and the DDF-SECSY with ultrafast acquisition scheme were applied under this field inhomogeneity. The conventional 2D SECSY experiments were performed with 1.5 s pulse repetition time and two transients, 1000×2219 points were acquired with spectral widths of 1800 Hz×3700 Hz (F1×F2) in 50 min. For the DDF-SECSY with ultrafast acquisition scheme, the width of a 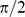 hard RF pulse was 10.5 µs. The width of the solvent-selective 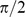 Gaussian pulse was 6.5 ms. The parameters of CSGs were the same as those used for the above experiment. The pulse repetition time was 1.0 s, the constant-time 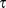 was 60 ms, and the echo time (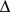) was 40 ms. The ultrafast acquisition parameters were set as follows: spatially encoded gradient 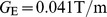 with a duration of 10 ms and spatially decoded gradient 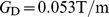 with a duration 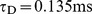, resulting in ultrafast dimensions (F2×F3) with spectral widths of 240 Hz×3700 Hz. Both spatial encoding and decoding gradients are applied along *z* direction. The F1 dimension with a spectral width of 1200 Hz was acquired with 600 *t*_1_ increments and four transients. The total acquisition time was 40 min.

## Results and Discussion

### Chemical solution

**Figure 3:**
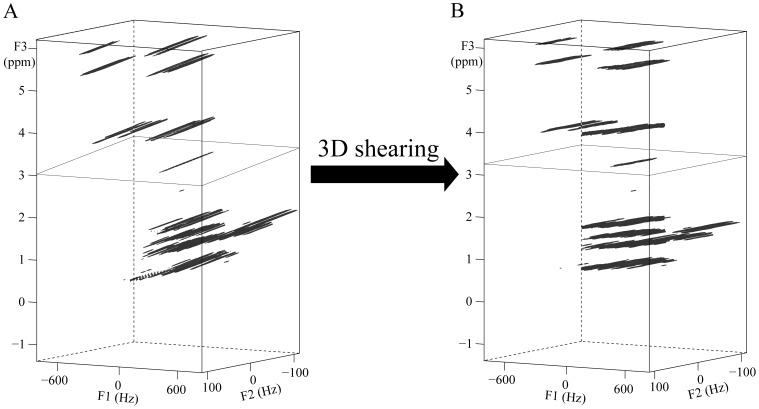
The shearing process for 3D DDF-SECSY data of butyl methacrylate in DMSO: (A) original 3D spectrum before shearing and (B) processed 3D spectrum after shearing. The given F1–F2 plane where a singlet signal at 3.1 ppm lies is selected to show the rotation of signal streaks by shearing process. Before shearing, the signal streaks are parallel to the F2–F3 plane and perpendicular to the F1 dimension. After shearing process, the signal streaks only stretch along F2 dimension and are perpendicular to the F1–F3 plane, resulting in high-resolution SECSY information on the F1–F3 plane.

**Figure 4:**
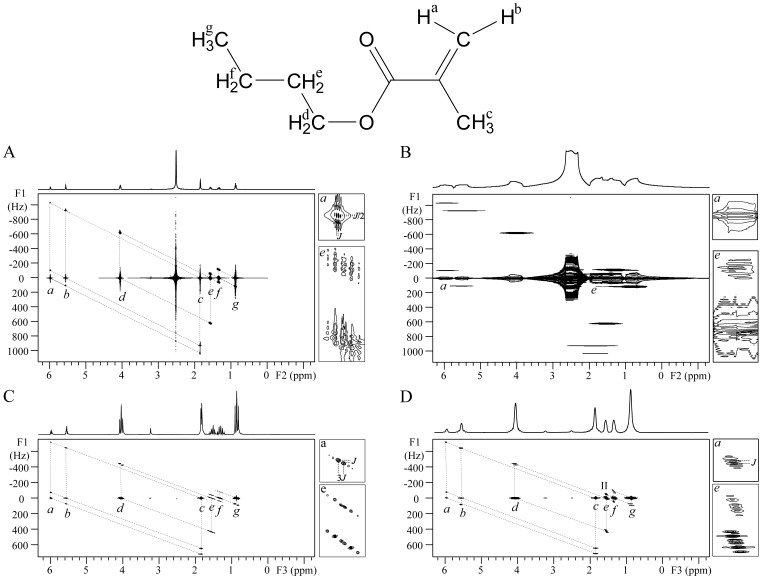
SECSY spectra of a solution of butyl methacrylate in DMSO. (A, B) Conventional spectrum with 1D projection along the F2 axis in a well-shimmed field (A) and in an inhomogeneous field with a line-width of 150 Hz (B); (C, D) spectra with 1D projection along the F3 axis in the same inhomogeneous field acquired from DDF-SECSY (C) and DDF-SECSY with ultrafast acquisition scheme (D). The regions of component peak of *a* and both component and cross peaks of *e* are expanded for comparison. The structure of butyl methacrylate with proton marked is shown on the top of the frame.

The results for a chemical solution of butyl methacrylate in DMSO are presented in Figs. 3 and 4. Firstly, the original 3D DDF-SECSY spectrum and the sheared 3D spectrum are given in Fig. 3 to demonstrate the shearing process for 2D DDF-SECSY spectrum. We can see from Fig. 3A that the original 3D DDF-SECSY suffers from inhomogeneous line broadening in the F2 and F3 dimensions but is free from inhomogeneous line broadening in the F1 dimension. The resulting signal streaks are parallel to the F2–F3 plane and perpendicular to the F1 dimension. After shearing the F2–F3 plane along the F3 axis, all the signal streaks only stretch along the F2 dimension and are perpendicular to the F1–F3 plane (Fig. 3B), which suggests the sheared 3D signals are free from the effect of field inhomogeneity along the F1 and F3 dimensions. In addition, the SECSY spectral information is observable in the F1–F3 plane. Therefore high-resolution 2D SECSY spectrum (Fig. 4C) can be obtained by projecting the 3D sheared spectrum onto the F1–F3 plane.

The 2D SECSY spectra from the proposed and conventional sequences are shown in Fig. 4. The molecular structure of butyl methacrylate is given on the top of Fig. 4, where seven different kinds of protons are marked by “*a*” to “*g*”. The singlet at 3.1 ppm is from impurity and not marked. The conventional SECSY spectrum acquired in a well shimmed magnetic field is shown in Fig. 4A. The standard SECSY spectral information is clearly presented and six pairs of coupled spins, *a* and *b*, *a* and *c*, *b* and *c*, *d* and *e*, *e* and *f*, and *f* and *g* protons, can be directly determined by matching parallelograms with their corresponding diagonals lying at F1 = 0 line. The regions marked by *a* and *e* in Fig. 4A are expanded to show the *J-*coupling multiplet patterns. The diagonal and cross peaks with complex multiplet patterns are shown in region *e*. The diagonal peaks with small *J* coupling are presented in region *a*, where the *J* splitting information can only be observed in the F2 dimension, and is not available in the F1 dimension due to the half-size of *J* coupling splitting in conventional SECSY spectrum. This small *J* coupling is measured in such a well shimmed field with effort and the value was measured to be 0.97 Hz. When a conventional SECSY spectrum was acquired in the inhomogeneous field, spectral information was destroyed by inhomogeneous line broadening (Fig. 4B). The *J*-coupling splitting become unresolved, and the adjacent peaks were overlapped along F1 = 0. It is hard to obtain useful information from the conventional SECSY spectrum acquired in the inhomogeneous field.

The high-resolution 2D SECSY spectrum obtained using the DDF-SECSY sequence in the same inhomogeneous field is shown in Fig. 4C. Note that the F1 spectral width in this 2D spectrum is 2/3 of that in conventional SECSY spectrum, and the solvent signal is suppressed. The information of chemical shift and multiplet patterns are preserved with high resolution and the peak overlap is avoided. Comparing the 1D projection spectra along the chemical shift dimension in Fig. 4B and C, we can see that the DDF-SECSY sequence has the ability to remove the influence of field inhomogeneity and the line-width is reduced from 150 Hz to 4 Hz. All the coupled spin systems can be analyzed according to the parallelogram networks marked with dotted lines in Fig. 4C, similar to the SECSY spectrum in Fig. 4A. From the expanded regions of *a* and *e*, straightforward multiplet patterns can be obtained. With the simplified spectral pattern, accurate analysis of *J* coupling becomes convenient. For example, it is easy to obtain the *J* coupling information from the diagonal and cross multiplets in the expanded regions. Even for the signal with small *J* coupling in the expanded region *a*, the *J* coupling multiplet is well resolved in both dimensions, and the accurate value of small *J* coupling can be achieved from the triple-magnified value along the F3 dimension, which is 3.1 Hz. It is clear that the 2D DDF-SECSY spectrum obtained from the inhomogeneous field is similar to the conventional 2D SECSY spectrum from the homogeneous field. Furthermore, the straightforward multiplet pattern and *J* coupling magnification in the 2D DDF-SECSY spectrum are useful for accurate analysis. It can be seen that there is a singlet peak located at (0 Hz, 3.24 ppm) in the 2D DDF-SECSY spectra (Fig. 4C and D). This singlet peak is from a non-*J* coupled spin and its resolution is the same with the resolution of peaks from *J* coupled spins. Hence, the DDF-SECSY sequence is also suitable for non-*J* coupled spin systems, which is different from the high-resolution method proposed by Pelupessy *et al.* [28].

The result of 2D DDF-SECSY with ultrafast acquisition scheme is shown in Fig. 4D. The acquisition efficiency was improved by 15, although with a cost. Due to the intrinsic resolution defect in ultrafast acquisition strategy, the spectral resolution in ultrafast dimension F3 remained 33 Hz, which hinders accurate determination of *J*-coupling constants. However, the spectral resolution in the F1 dimension was improved from 150 Hz to 6 Hz. It can be seen from the expanded regions *a* and *e* that the *J* coupling information can be obtained along the F1 dimension. The information of coupled spin networks can also be obtained from Fig. 4D. Hence, the DDF-SECSY provides a way for the SECSY detection in inhomogeneous fields. Two options can be selected according to different requirements. For accurate measurements such as in chemical analysis, the DDF-SECSY sequence without ultrafast acquisition scheme is more suitable for high-resolution information, whereas for the measurements when time is a concern, the ultrafast acquisition scheme can be introduced by trading off spectral resolution and signal intensity.

### Brain phantom

**Figure 5:**
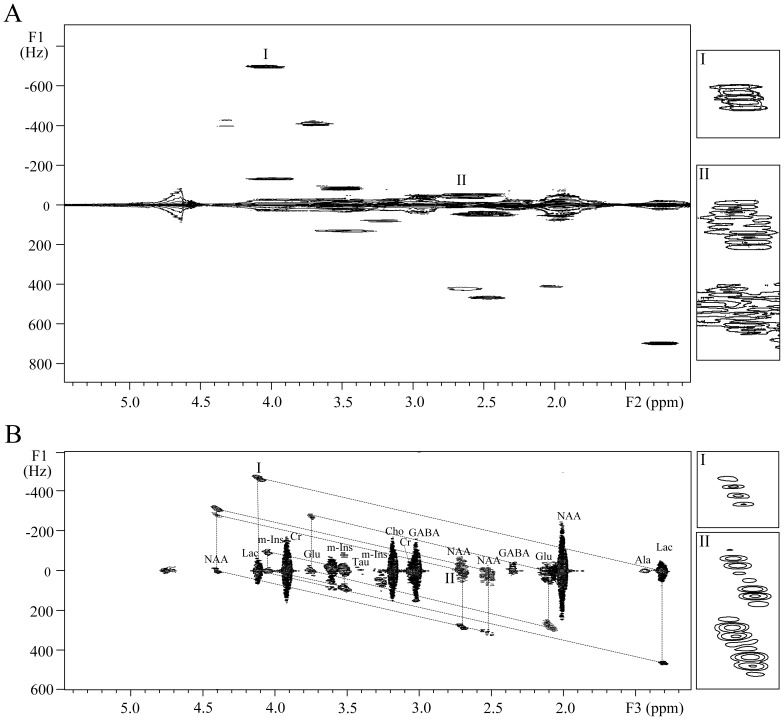
SECSY spectra of an aqueous solution of brain metabolites. (A) Conventional spectrum in an inhomogeneous field with a line-width of 200 Hz; (B) DDF-SECSY spectrum using ultrafast acquisition scheme. The marked regions are expanded for comparison.

The experimental results of the brain phantom are presented in Fig. 5. For the detection of brain metabolites in practice, the identification and correlation assignment of metabolites, related to some disease diagnosis and recognition of metabolic pathway, are more interesting than the accurate analysis of molecular structure of a given metabolite for which high spectral resolution is generally required. Hence, the DDF-SECSY with ultrafast acquisition scheme was applied in this detection. In the conventional SECSY spectrum shown in Fig. 5A, the spectral information suffers from inhomogeneous field effect along the F1 dimension. The metabolite assignment is blocked by the overlap of signal peaks along F1 = 0, and the coupled correlation information of metabolites becomes ambiguous due to the extremely low resolution in the F1 dimension. The DDF-SECSY provides a solution for resolution enhancement within reasonable acquisition time (Fig. 5B). The spectral resolution along the F3 dimension of DDF-SECSY spectrum was improved from 200 Hz to 31 Hz, and the strong water signal was suppressed. Although the line-width is still 31 Hz in the F3 dimension due to the intrinsic resolution defect in ultrafast acquisition, signal assignment is workable and nine brain metabolites could be identified and assigned from the spectrum in Fig. 5B: Lac, Ala, NAA, Glu, GABA, Cr, Cho, m-Ins, Tau. Seven pairs of coupled spins were found and marked by dotted-line parallelograms. For example, the peaks of -CH_3_ (0 Hz, 1.31 ppm) and -CH (0 Hz, 4.11 ppm) are found for Lac (CH_3_-CH(OH)-COOH) along F1 = 0. Additionally, the *J* coupling of CH_3_-CH is visualized from the cross peaks at (466.67 Hz, 1.31 ppm) and (−466.67 Hz, 4.11 ppm) in related dotted-line parallelogram. Similarly, the coupled information for NAA, Glu, and m-Ins could also be found. In addition, the information for non-*J* coupled spins of metabolites could also be found, i.e. peak located at (0 Hz, 1.99 ppm) is from non-*J* coupled spins of NAA, (0 Hz, 3.05 ppm) is from non-*J* coupled spins of Cr. Although the spectral resolution was low for the *J* coupling detection along the F3 dimension, the *J* coupling constants were detectable along the F1 dimension as seen from the expanded regions I and II in Fig. 5B.

## Conclusions

In this paper, a new pulse sequence named DDF-SECSY was proposed based on the DDF modulation and 3D acquisition for high-resolution 2D SECSY in inhomogeneous fields. Besides providing an improvement in spectral resolution, new and straightforward multiplet patterns for *J* coupling detection were obtained that are useful for accurate spectral analysis. Based on the consideration of spectral resolution and acquisition efficiency, two different acquisition options were provided: the normal acquisition mode with longer acquisition time for more accurate measurement, such as in a chemical analysis, or the ultrafast acquisition scheme for detection with higher time efficiency, such as when complicated metabolites measurement. The DDF treatment combined with the reduced product operator formalism was applied to derive theoretical expression for the 3D signal. Our experimental observations were consistent with our theoretical predictions. The results reveal that the new sequence is an attractive way to eliminate the inhomogeneous line broadening in the SECSY spectrum and provides a promising tool for the applications of metabolite detection in metabonomics, even for the applications on *in vivo* and *in situ* samples. Since the signal intensity from the new sequences is still lower than that of conventional SQC signals, improving the new sequences for practical applications may be achieved by combination with the dynamic nuclear polarization (DNP) technique [29], which is being actively explored.
